# Mailing human papillomavirus self-sampling kits to women under-screened for cervical cancer improved detection in cervical cancer screening in a general population study in Japan

**DOI:** 10.1186/s12889-023-15402-7

**Published:** 2023-03-11

**Authors:** Yoko Nishimura, Motoki Matsuura, Noriko Terada, Sachiko Nagao, Hiroshi Shimada, Kyoko Isoyama, Masato Tamate, Masahiro Iwasaki, Tsuyoshi Saito

**Affiliations:** 1 Department of Obstetrics and Gynecology, Sapporo Medical University, S1 W17, Chuo-Ku, Sapporo, 060-8543 Japan; 2 Department of Obstetrics and Gynecology, Steel Memorial Muroran Hospital, Muroran, Japan; 3 Department of Obstetrics and Gynecology, Nikko Memorial Hospital, Muroran, Japan

**Keywords:** Cervical cancer, Human papilloma virus, Self-collected sample, Under-screened women

## Abstract

**Background:**

One cause of the increase in cervical cancer rates in Japan is the long-term stagnation in the cervical cancer screening consultation rate. Therefore, improving the screening consultation rate is of urgent concern to reduce cervical cancer incidence. Self-collected human papilloma virus (HPV) tests have been successfully adopted in several countries, such as the Netherlands and Australia, as a measure of individuals who have not undergone cervical cancer screening in national programs. This study aimed to verify whether self-collected HPV tests presented an effective countermeasure for individuals who had not undergone the recommended cervical cancer screenings.

**Methods:**

This study was conducted from December 2020 to September 2022 in Muroran City, Japan. The primary evaluated endpoint was the percentage of citizens who underwent cervical cancer screening at a hospital with positive self-collected HPV test results. The secondary endpoint was the percentage of included participants who were diagnosed with cervical intraepithelial neoplasia (CIN) or higher among those who visited a hospital and underwent cervical cancer screening.

**Results:**

The included study participants were 7,653 individuals aged 20–50 years with no record of previous cervical cancer examination in the past 5 years. We mailed these participants information on self-administered HPV tests as an alternative screening procedure and sent the kit to 1,674 women who requested the test. Among them, 953 returned the kit. Among the 89 HPV-positive individuals (positive rate, 9.3%), 71 (79.8%) visited the designated hospital for an examination. A closer examination revealed that 13 women (18.3% of hospital visits) had a CIN finding of CIN2 or higher, among whom one each had cervical cancer and vulvar cancer, eight presented with CIN3, and three presented with CIN2; two cases of invasive gynecologic cancer were also identified.

**Conclusions:**

We conclude that the self-collected HPV tests showed a certain efficacy as a measure of individuals who had not undergone the recommended cervical cancer screening. We devised ways to have the unexamined patients undergo HPV testing and ensure that HPV-positive individuals visited the hospital. Despite a few limitations, our findings suggest the effectiveness of this public health intervention.

## Background

Cervical cancer is highly preventable if cervical dysplasia is detected and treated before it becomes an invasive cancer. In 2020, 604,127 and 12,785 women were diagnosed with cervical cancer, and approximately 341,831 and 4,213 women died from the disease worldwide and in Japan, respectively [1]. One cause of the increase in the prevalence of cervical cancer in Japan is the long-term stagnation of the cervical cancer screening consultation rate in this country [2]. Therefore, improving the screening consultation rate is an urgent public health issue concerning the reduction of cervical cancer incidence. The World Health Organization (WHO) has stated that the following targets must be met by 2030 for countries to be on the path toward the elimination of cervical cancer: 90% of girls must be fully vaccinated with the human papilloma virus (HPV) vaccine by the age of 15 years, 70% of women must be screened with a high-performance test by the age of 35 years and again by the age of 45 years, and 90% of women identified as having cervical cancer and precancerous lesions must receive treatment [3].

In Japan, the target rate for cervical cancer screening is set at 50% or higher. However, the effective rate is currently approximately 40%, which places Japan within the lowest categorization for screening utilization among the Organization for Economic Co-operation and Development countries [4]. The reasons for non-participation in screening programs likely include lack of time, inconvenience, shame, pain, and cost [5, 6]. To increase the number of women undergoing medical examinations, it is necessary not only to improve the healthcare environment and the convenience of screening but also to actively encourage women who have not yet undergone medical examinations to participate in screening programs.

A recent meta-analysis encompassing 37 studies and enrolling a total of 18,516 women from 24 countries across five continents indicated strong acceptance of self-sampling interventions as well as a preference for self-sampling over clinician sampling [7]. Moreover, studies from many countries, such as the Netherlands, Mexico, France, United Kingdom, Finland, Sweden Argentina, Italy, Norway, Australia, and Switzerland (including studies conducted on the national level as well as those focusing specific socioeconomic groups), have demonstrated that offering self-sampling can lead to increased participation rates in cervical cancer screening programs [8]. In several countries, including the Netherlands and Australia, self-collected HPV tests have been successfully adopted as a measure of individuals who have not undergone the recommended cervical cancer screening in national programs [8].

HPV testing using self-collected cervical and urine samples is convenient as well as effective for improving the screening rate. However, to serve as a viable alternative screening technique, this intervention must have a sensitivity equivalent to HPV testing using physician-collected cervical samples. Our research group had previously reported that HPV testing results using self-collected cervical and urine samples were consistent with those obtained from physician-collected samples [9]. HPV testing using self-collected samples and urine samples appears to be a viable means of screening for cervical cancer in women who would otherwise not undergo HPV testing [9].

In the present study, we verified whether self-collected HPV testing was effective as a countermeasure for individuals who had not yet undergone cervical cancer screening. We conducted this study with the cooperation of the Health and Welfare Department, Health Promotion Division of Muroran City in Hokkaido, Japan. We hypothesized that many participants who had been identified as HPV positive would visit the hospital, and those with positive screening results would be more likely to present with cervical cancer or cervical dysplasia (i.e., precancerous lesions) on closer examination.

## Methods

This study was approved by the ethical review board of Sapporo Medical University (312–87, 2019/08/08, Sapporo, Japan) and was conducted in accordance with the principles of the Declaration of Helsinki. The privacy and anonymity of the participants were maintained. Written informed consent was obtained from all participants.

Self-collected cervical sampling was performed using Evalyn Brush® (HARADA, Osaka, Japan). The procedure for sampling using this brush involves inserting the brush casing as far as possible into the vagina, following which the brush is pushed out, rotated five times, and then removed from the vagina with the brush still pushed out. This accomplishes effective swabbing of the entire vagina for cervical cancer testing. For the urine samples, 18–22 mL of first-catch urine was collected using a COLLI-PEE® kit (Nonosanis, Wijnegem, Belgium).

The samples were tested for HPV with the Cobas® 8800 system (Roche Diagnostics, Inc., Basel, Switzerland). HPV test results were divided into type 16, type 18, and other high-risk types (HPV types 31, 33, 35, 39, 45, 51, 52, 56, 58, 59, 66, and 68).

This research was conducted in Muroran City (Hokkaido, Japan), which was selected as the site of the current study because there are only three general hospitals in Muroran City that offer gynecological outpatient clinics, and it takes more than 1 h to visit a gynecologist in another city for those residing outside Muroran City. Moreover, almost all people who test positive on routine screenings visit these gynecological outpatient clinics. Therefore, accurate consultation rates and follow-ups are possible.

The Health and Welfare Department, Health Promotion Division of Muroran City, searched for residents who had not undergone cervical cancer screening between December 2015 and November 2020. The present study targeted 7,653 women aged 20–50 years for the evaluated intervention. We mailed information regarding the screening intervention to 7,653 women who had not undergone the recommended cervical cancer examination within the past 5 years. The applicant was asked to complete a consent form, including their name and address, and to reply to the Department of Obstetrics and Gynecology at Sapporo Medical University if they were interested in participating in this screening program. It was stated that the investigator would decide whether to send a self-collection HPV test or urine collection set.

Then, the self-collected HPV test kits were mailed to interested participants. At this time, we enclosed an explanation of the study, including its associated risks and benefits, along with a separate consent form. Applicants mailed the kit and consent form to the Department of Obstetrics and Gynecology at Sapporo Medical University. Then, the kits were anonymized and submitted to the abovementioned external inspection company (LSI Medience, Tokyo, Japan), which notified the designated obstetrics and gynecology department of the testing results. Then, the obstetrics and gynecology department mailed the HPV testing results to each participant and recommended that those who tested positive for HPV should visit a designated hospital for follow-up. The gynecologist at the designated medical institution notified the physicians at the Department of Obstetrics and Gynecology at Sapporo Medical University of the findings of the cytological and histological examinations and asked these physicians to inform the participants of the follow-up testing results.

The participants who underwent the test also completed a questionnaire. The questionnaire consisted of the following five items: (i) how did you feel about the free HPV test? (ii) how was the test method? (iii) do you know about HPV? (iv) what do you think about the HPV vaccine? and (v) if it was a paid test, how much would it cost in Japanese yen?

The primary evaluated endpoint was the percentage of citizens who underwent cervical cancer screening at a hospital with positive self-collected HPV test results. The secondary endpoint was the percentage of included participants diagnosed with cervical intraepithelial neoplasia (CIN) or higher among those who visited a hospital and underwent cervical cancer screening.

The age difference between those with CIN2 or higher and those with CIN1 or lower was examined using a t-test. Pearson’s chi-square test was used to examine the differences in screening history and delivery history between those with CIN2 or higher and those with CIN1 or lower. Two-sided *P*-values of < 0.05 were considered statistically significant. All analyses were conducted using SPSS statistical software (IBM Corp., Armonk, NY, USA).

## Results

Although information was sent to 7,653 participants, 88 of them did not receive the information because their recorded addresses were invalid. Therefore, the information was effectively sent to a total of 7,565 participants. Among those individuals, 1,674 women requested for the test on receipt of information regarding the study intervention, submitted a research consent form to our university, and were mailed the sampling kit. There were 23 women who responded that they would not use the self-collection kit but would use the urine collection kit. Therefore, we sent the urine collection kit to them. The total percentage of respondents for either screening test was 22.1%.

**Fig. 1:**
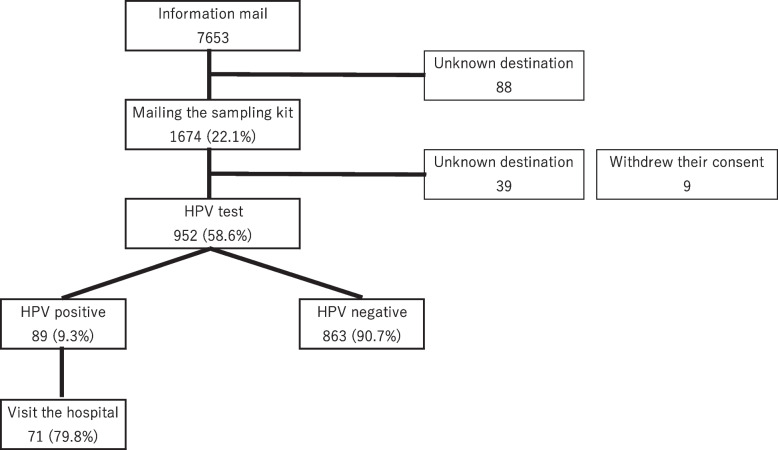
Study flowchart

After the kit was sent, 39 participants did not receive it owing to an invalid address and 9 participants withdrew their consent. A total of 953 participants returned the kit, although one had a flawed consent form; 952 kits were ultimately submitted for inspection, and 58.6% of those who mailed the kit agreed to HPV testing (Fig. 1).

We identified 89 HPV-positive individuals in this study (positive rate, 9.3%), and 71 of the HPV-positive individuals (79.8%) visited the designated hospital for an examination (Fig. 1). Among the HPV-positive individuals, the breakdown according to HPV type was as follows: *n* = 14, 4, and 77 for type 16, type 18, and other HPV types, respectively; 4 of the 77 participants with “other” HPV types were co-infected with HPV type 16 (Table 1).

**Table 1:** The breakdown of HPV types among the HPV-positive individuals

	n
HPV 16	14
HPV 18	4
HPV Other	77^a^

^a^Four of the 77 were duplicated with type 16

**Fig. 2:**
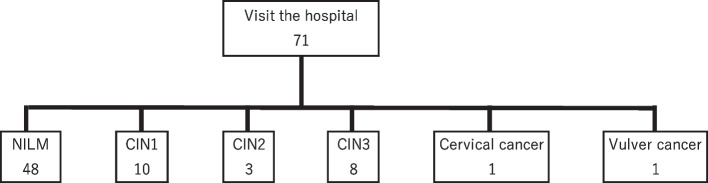
Cytology and histology results of women visiting the hospital for testing

A closer examination revealed that 13 participants (18.3%) had a CIN finding of CIN2 or higher. Specifically, the breakdown of diagnostic findings was as follows: one for cervical cancer, one for vulvar cancer, eight for CIN3, and three for CIN2 (Fig. 2). This study detected two cases of invasive gynecologic cancer. Among participants presenting with CIN2 or higher, the breakdown by HPV type was as follows: 5 for type 16 and 10 for other HPV types; 2 of the 10 participants with “other” HPV types were co-infected with type 16. Among the participants who opted for the urine collection kit, only one tested HPV-positive and had a normal cytology at hospital visit.

Demographic data were available for 71 individuals who underwent a detailed examination; these data are presented in Table 2. The average age of these participants was 36.3 ± 8.7 years. Moreover, among the 71 participants, 29 had never undergone a cervical cancer examination, 13 had not been examined for more than 5 years, and 29 had been examined within 5 years. The city most likely did not have the last category of individuals recorded as having undergone examinations because these 29 people had undergone private medical examinations (i.e., not the medical examinations provided by the city or cervical cancer tests performed early in pregnancy).

**Table 2:** The demographic data for 71 individuals who underwent a detailed inspection

Age (mean ± SD)	36.3 ± 8.7	
CIN 2 or higher	41.8 ± 7.0	*p* = 0.00338
CIN 1 or lower	35.0 ± 8.7	
The actual situation		
Truly unexamined (n)	29	
Unexamined for more than 5 years (n)	13	
Examined within 5 years (n)	29	

The average age of the participants presenting with findings of CIN2 or higher was 41.8 ± 7.0 years, and the average age of those presenting with findings of CIN1 or below was 35.0 ± 8.7 years. Those presenting with CIN2 or higher were significantly older on average than those presenting with findings of CIN1 or below (*p* = 0.00338).

Moreover, on examining the proportion of patients who had never undergone cervical cancer screening, there was no significant difference detected between those with findings of CIN2 or higher (*n* = 5, 38.5%) or CIN1 or below (*n* = 24, 41.4%; *p* = 0.8466, Table 3). On examination of delivery history, seven (53.8%) participants had a history of delivery with a finding of CIN2 or higher, and 24 (41.4%) had a history of delivery with a finding of CIN1 or below (*p* = 0.4127, Table 3).

**Table 3:** The demographic detail data by diagnosis

	n (%)	*p*
The proportion of true unexamined patients		
CIN 2 or higher	5 (38.5)	*p* = 0.8466
CIN 1 or lower	24 (41.4)	
Delivery history		
CIN 2 or higher	7 (53.8)	*p* = 0.4127
CIN 1 or lower	24 (41.4)	

On evaluating the age distribution in those with findings of CIN2 or higher or CIN1 or below, the percentages of those aged 20–29, 30–39, and 40–50 years were 7.7% (*n* = 1) and 36.2% (*n* = 21), 23.1% (*n* = 3) and 27.6% (*n* = 16), and 69.2% (*n* = 9) and 36.2% (*n* = 21), respectively (Table 4). The rate of being diagnosed with CIN2 of higher was significantly lower for those in their 20 s even if their HPV findings were positive (*p* = 0.0445), whereas the rate of being diagnosed with CIN2 or higher was significantly higher in those aged 40–50 years (*p* = 0.0294).

**Table 4:** The percentages by age in the group with over CIN 2 or under CIN 1

	CIN 2 or higher % (n)	CIN 1 or lower % (n)	*p*
20–29 years old (%)	7.7 (1)	36.2 (21)	0.0445
30–39 years old (%)	23.1 (3)	27.6 (16)	0.7399
40–50 years old (%)	69.2 (9)	36.2 (21)	0.0294

**Fig. 3:**
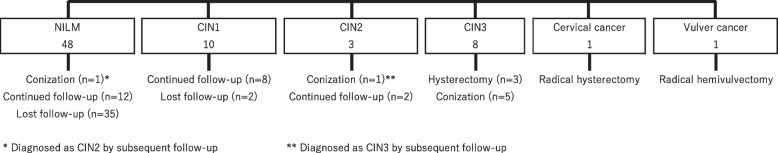
Post-testing follow-up status and outcomes

The participants’ subsequent follow-up status and outcomes are shown in Fig. 3. The mean follow-up time was 8.6 months. Two women diagnosed with cervical cancer and vulvar cancer underwent radical surgery. Among the eight participants diagnosed with CIN3, three underwent total hysterectomy and five underwent cervical conization. Among the three participants diagnosed with CIN2, one was diagnosed with CIN3 at a subsequent follow-up visit and underwent cervical conization.

Of the three participants diagnosed with CIN2, two continue to visit the designated hospital at the time of reporting this study. Of the 10 women diagnosed with CIN1, eight continue to visit the hospital and two subsequently stopped visiting the hospital. It remains unknown whether they have been admitted to other hospitals since then. Of the 48 women found to test negative for intraepithelial lesion or malignancy, 12 continue to visit the hospital; one was diagnosed with CIN2 at a subsequent follow-up visit and underwent cervical conization. Of the 48 women diagnosed with normal cytology, 35 stopped visiting the designated hospital (Fig. 3). This study found that a total of 12 women underwent surgery following a diagnosis of a precancerous lesion or early cancer.

**Fig. 4:**
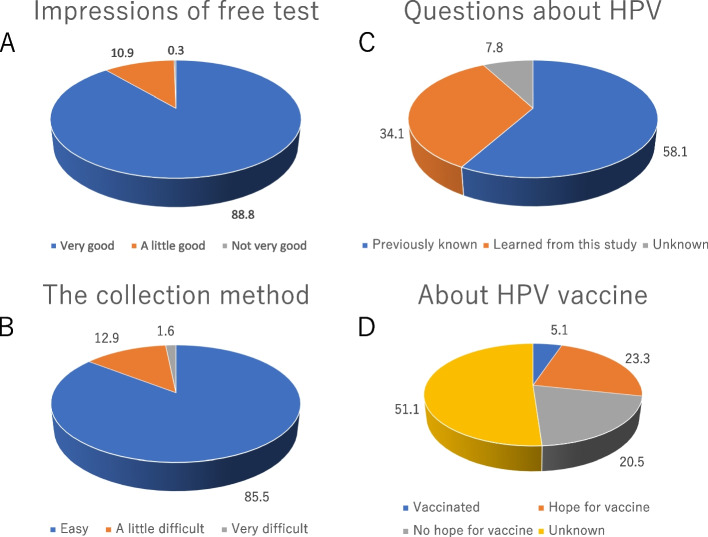
Findings of the study questionnaire. **A** Impressions of free test, **B** The collection method, **C** Questions about HPV, **D** About HPV vaccine

**Fig. 5:**
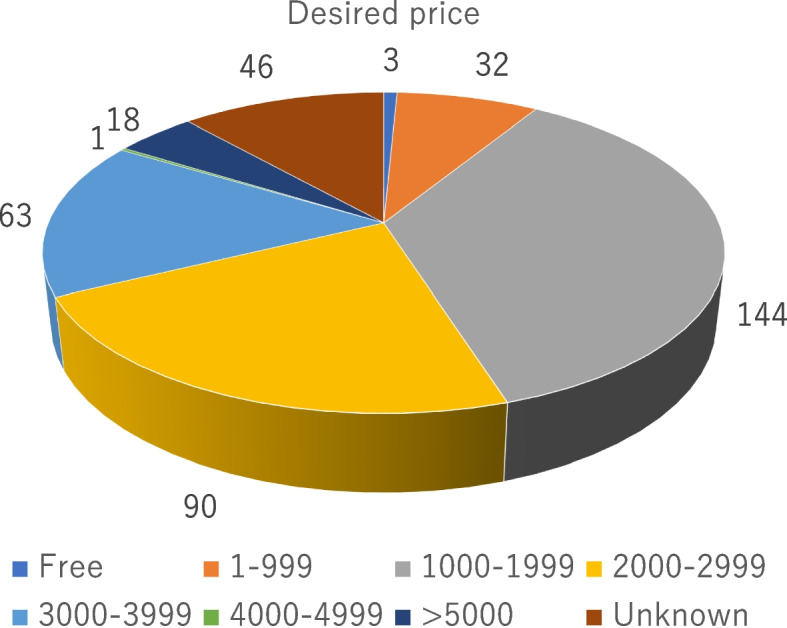
Cost structure of the evaluated intervention

Among participants who completed the self-collected cervical or urine collection tests, 397 responded to the study questionnaire. The results of this questionnaire are presented in Fig. 4. A total of 88.8% of the survey participants responded that this free testing intervention was “very good,” whereas 10.9% answered that it provided only a slight benefit (Fig. 4A). A total of 85.5% of the respondents responded that the collection method was easy, whereas 12.9% responded that it was somewhat challenging (Fig. 4B). When asked about HPV, only 58.1% had previously known that HPV was the causative virus for cervical cancer, and 34.1% stated that they became aware of the causative virus (i.e., HPV) only after participating in this study (Fig. 4C). Among the individuals participating in this study, only 5.1% were vaccinated with the HPV vaccine, although 23.3% stated that they would like to receive the vaccine in the future. However, 20.5% of the participants responded that they did not wish to receive the vaccine (Fig. 4D). We note that the median cost of the evaluated intervention was 1,500 yen (range, 0–10,000 yen) (Fig. 5).

## Discussion

Cervical cancer is highly preventable and, therefore, improving the screening consultation rate is an urgent issue for the reduction of the prevalence of cervical cancer. We researched whether a self-collected HPV test was effective as a countermeasure for those who had not yet undergone the recommended cervical cancer screening. In this study, 12.6% of individuals who were initially referred were tested, and 1.4% of those who were tested were able to undergo surgery for invasive cancer or precancerous lesions. Our findings revealed that the self-collected HPV test showed certain efficacy as a screening measure for evaluating those who had not yet undergone traditional cervical cancer screening. Those who had not undergone cervical cancer screening and presented with positive HPV findings were encouraged to undergo a closer examination at a designated hospital. Our results suggest that it may become an effective method in the future in Japan, where the screening rate is low and cervical cancer is on the rise. Moreover, our results suggest the importance of recommendations on follow-up testing for HPV-positive individuals.

In Japan, the rate of cervical cancer screening is low and the active recommendation for vaccination against cervical cancer has been suspended for some time [2]. Therefore, improving the rate of cervical cancer screening is a critically important public health issue. We conducted this study because self-collected HPV tests have already been introduced and shown to be effective in these countries. Moreover, we thought that this intervention would be effective in guiding women at a high risk of HPV positivity (rather than recommending traditional cytopathological examinations to the unexamined patients).

We conclude that HPV testing using self-collected samples is a convenient and effective means for improving the cervical cancer screening rate. However, to serve as an alternative cervical cancer screening technique, this method must show a sensitivity equivalent to that of HPV testing using physician-collected cervical samples.

We previously reported that the results were consistent between physician- and self-collected samples and that the agreement rate was especially high for HPV types 16 and 18 (as measured using κ-values) [9]. Among women presenting with CIN3, when a positive physician-collected sample was considered a true positive, the sensitivity and specificity of self-collected samples were 100% and 100% for HPV type 16, 75% and 100% for HPV type 18, and 92.0% and 96.3% for the other HPV types, respectively [9].

Onuma et al. reported that HPV self-sampling results showed good agreement with physician sampling results and that the sensitivity of CIN2 detection by self-sampling was as high as that of physician sampling [10]. Similarly, Ngu et al. reported that the concordance rate for HPV detection between self-sampled and clinician-sampled specimens was 90.2% (95% confidence interval [CI]: 85.1–93.8%; Cohen’s kappa 0.59 [95% CI: 0.42–0.75]) in Hong Kong. These researchers conclude that HPV self-sampling is an effective method for cervical cancer screening and can be considered an effective screening option, especially in women who are reluctant or unable to attend regular screenings [11].

In addition, Saville et al. reported that HPV16/18 results showed high observed agreement between self- and practitioner-collected samples in all assays (range: 0.94–0.99), with good agreement for non-HPV16/18 oncogenic HPV types (range: 0.64–0.73). These researchers concluded that self-collection showed good concordance and sensitivity for HPV-based cervical screening compared to those for practitioner-collected samples in assays conducted within the Australian National Cervical Screening Program [12]. These results indicate that self-administered HPV tests may be a viable option for cervical cancer screening.

Several studies have reported that self-collected HPV tests have led to an increase in the consultation rates [13–16]. For example, in a study conducted in Sweden, Sanner et al. reported that 63% of the women invited to participate in their study (i.e., those who did not have a screening record within the previous 6 years) requested a self-testing kit and that 39% of these women sent a self-sampling kit to the designated laboratory [13]. In another study conducted in Copenhagen, Denmark, Lam et al. reported that 32% of the recruited women who were not screened in the previous 4–6 years requested self-sampling kits and that 20% sent a self-sampling kit to the designated laboratory [14].

Moreover, Arbyn et al. examined the acceptance rate of self-collecting HPV tests in a randomized controlled trial [15]. The mean percentage of women in the self-sampling arm with an HPV test conducted on a self-sample after the research team mailed a self-sampling kit to the participant’s home in a “mail-to-all” campaign was 19.2% (95% CI: 15.7%–23.0%) [15]. The pooled participation rate was 7.8% (95% CI: 5.2%–10.9%) when women had to request a self-sampling kit (i.e., opt-in) [15]. The pooled percentage of participating women in the intention-to-treat analysis within the self-sampling arm was 17.7% (12.3%–23.9%) in the opt-in scenario [15]. These researchers concluded that offering self-sampling kits was generally more effective in reaching under-screened women than sending invitations. Moreover, these researchers reported follow-up compliance rates for HPV-positive individuals in a meta-analysis evaluating a self-collected HPV testing intervention, with a consultation rate of 70.4% (95% CI: 58.3%–81.3%) in reference to cytological triage [15]. The corresponding consultation rate in our study was 79.8%, which was higher than that of the prior meta-analysis. The high consultation rate in our study may be attributed to the designation of our hospital for follow-up visitation and testing.

The detection rate for presentation of CIN2 or higher was as high as 18.3% on cytology triage of the HPV-positive individuals enrolled in this study. When screening using cytology alone, the detection rate for presenting CIN2 or higher was reported as being approximately 4.4% in the literature [17]. The corresponding detection rate was higher in the present study. However, our study found two cases of invasive gynecologic cancer, whereas no patients with cervical cancer were diagnosed within the standard cervical cancer screening performed in Muroran City during the same time period. This study confirmed that the HPV-positive cases who had not undergone cervical cancer screening were at high risk of cervical cancer.

Our study has some limitations. First, it is currently unclear which ages should be recommended for completing self-administered HPV kits. For example, there is currently no clear consensus as to whether women aged > 20 years should be contacted or if the targeted age group should be those aged > 30 years. Moreover, the upper age limit for this intervention remains unclear. Including women of younger age may lead to the detection of transient HPV infections as well as presenting a cause of unnecessary anxiety. In fact, our study revealed that the rate of being diagnosed with a finding of CIN2 or higher was significantly lower in those in their 20 s given positive HPV findings. However, to widely recommend medical examinations to the unvaccinated generation, it is necessary to inform the younger generation of this critically important public health concern and initiative.

Second, there was an unclear optimal subsequent follow-up period for women who were found to be HPV-positive. Although it is sufficient for the participants to visit the hospital regularly following the detection of positive HPV findings, there is a possibility that a woman will elect not to undergo examinations. Therefore, effectively managing post-testing follow-up is of critical importance. Moreover, the specific testing that should be provided to individuals who have been diagnosed as HPV-positive remains unclear. Options include colposcopy as well as cytology prior to colposcopy. It may be possible to triage testing strategies according to the HPV type. For example, while colposcopy may be performed for HPV type 16, it may be possible to evaluate cytology prior to performing colposcopy for other HPV types.

Third, this study included women who were not truly unexamined (i.e., who had been examined for HPV/cervical cancer more than 5 years prior as well as those who had undergone screenings elsewhere). We conclude that it is necessary to evaluate the status of the city's medical examination system in the future. It is also necessary to publicize the reliability of the evaluated screening intervention, as some citizens may not have participated in this study because they did not trust the self-collected HPV testing method.

Fourth, we conducted an opt-in study. It may be preferable to send the kit only to those who request it, send the kit to all the unexamined people, or distribute a coupon for testing at the city facility. This remains to be elucidated in future studies. Two previous meta-analyses have revealed that sending self-collection kits directly to the participants’ home address generated a higher response rate than sending an invitation letter [18, 19]. How to best send self-collection kits to at-risk citizens is a critical issue to resolve within future investigations.

The strength of this study is that many of the patients who had not been examined had an opportunity to visit the hospital and that several patients successfully underwent treatment for cervical cancer or precancerous lesions through surgery. This strategy should be useful for health management in the future because most participants continue to follow through after undergoing the test.

## Conclusions

We showed that the self-collected HPV tests evaluated in the present study showed certain efficacy as a public health measure of those who had not yet undergone cervical cancer screening. Moreover, we devised a method for encouraging unexamined patients to undergo HPV testing and to ensure that HPV-positive individuals visited the hospital for follow-up testing; however, some issues remain to be resolved in the future, such as selecting among various countermeasures and two patients being diagnosed with malignant cancer and several more found to require surgery. Therefore, we would like to highlight the demonstrated effectiveness of this public health intervention. Future studies will help identify the optimal strategy for increasing the cervical cancer screening rates and efficacy.

## Data Availability

The data used for this study, though not available in a public repository, will be made available to other researchers upon reasonable request. The datasets used and analyzed during the current study are available from the corresponding author on reasonable request.

## Abbreviations

CIN: Cervical intraepithelial neoplasia
HPV: Human papilloma virus
WHO: World Health Organization
